# Culture counts: the diverse effects of culture and society on mental health amidst COVID-19 outbreak in Australia

**DOI:** 10.1017/ipm.2020.37

**Published:** 2020-05-14

**Authors:** Y. Furlong, T. Finnie

**Affiliations:** 1 Child and Adolescent Mental Health Service, Perth Children’s Hospital, Nedlands, WA 6909, Australia; 2 Centre and Discipline of Child and Adolescent Psychiatry, Psychosomatics and Psychotherapy, The University of Western Australia, Crawley, WA 6009, Australia; 3 UWA's Business School, The University of Western Australia, Crawley, WA 6009, Australia

**Keywords:** Australia, COVID-19, cultural competency, mental health, psychoanalysis

## Abstract

Since COVID-19 first emerged internationally, Australia has applied a number of public health measures to counter the disease’ epidemiology. The public heath response has been effective in virus testing, diagnosing and treating patients with COVID-19. The imposed strict border restrictions and social distancing played a vital role in reducing positive cases via community transmission resulting in ‘flattening of the curve’. Now is too soon to assess the impact of COVID-19 on people’s mental health, as it will be determined by both short- and long-term consequences of exposure to stress, uncertainty, loss of control, loneliness and isolation. The authors explored cultural and societal influences on mental health during the current pandemic utilising Geert Hofstede’s multidimensional construct of culture and determined psychological and cultural factors that foster resilience. We also reflected on the psychological impact of the pandemic on the individual and the group at large by utilising Michel Foucault’ and Jacques Lacan’ psychoanalytic theories. Remote Aboriginal Australian communities have been identified as a high-risk subpopulation in view of their unique vulnerabilities owing to their compromised health status, in addition to historical, systemic and cultural factors. Historically, Australia has prided itself in its multiculturalism; however, there has been evidence of an increase in racial microaggressions and xenophobia during this pandemic. Australia’s model of cultural awareness will need to evolve, from reactionary to more reflective, post COVID-19 pandemic to best serve our multicultural, inclusive and integrated society.

Life in Australia has changed dramatically in recent weeks due the COVID-19 pandemic. The impact upon how we live, work, socialise and communicate is stark in comparison to pre-COVID-19. The coronavirus outbreak was so sudden in its impact, and we are still attempting to grasp who is most at-risk and who may live or die from this acute respiratory syndrome. To date, the global outcome amounts to over three million confirmed cases resulting in 238 628 deaths worldwide at the time of writing (WHO, 2020). New evidence may explain these deadly figures, as it emerged that the virus has diversified into multiple lineages (Eden *et al*. 2020) and has neuro-invasive potential to cause respiratory failure due to the involvement of neurons in the medulla oblongata that controls involuntary respiration (Li *et al*. 2020; Mao *et al*. 2020).

## Australian response

Since COVID-19 first emerged internationally, Australia has implemented a number of public health measures to counter the disease’s epidemiology and for the first time utilised the powers under the Biosecurity Act’15 (Maclean & Elphick, 2020). The bulk of COVID-19-positive cases were imported by people travelling from overseas (63.4%), as well as acquired on board of cruise ships (17.6%) docking in Australian ports (Department of Health, 2020). Federal government imposed first quarantine restrictions on 29 January, 2 weeks after the first confirmed case, for travellers from China. These measures were widened to include all returning overseas travellers and eventually resulted in an international travel ban. Subsequent measures were employed at domestic level prohibiting social gatherings with the closure of most public facilities. The funeral arrangements have been limited to no more than 10 people (Office of PM, 2020*a*), while traditional Aboriginal funerals can number hundreds of First Nation people proudly observing their collective expression of grief in a so-called ‘sorry business’ ritual. Based on the latest low mortality rate of 95 deaths (out of 6801 confirmed cases), the public heath response has been effective towards virus testing, diagnosing and treating patients with COVID-19. The imposed strict border restrictions and social distancing played a vital role in reducing positive cases via community transmission resulting in ‘flattening of the curve’ (Department of Health, 2020).

Now it is too soon to make assumptions on the impact of COVID-19 on people’s mental health, as it will be determined by both short- and long-term consequences of exposure to stress, uncertainty, loss of control, loneliness and isolation. Prior to the pandemic, a survey from VicHealth reported that one in three people aged 18 to 25 years described problematic levels of loneliness (Victorian Health, 2019); this vulnerable group may be particularly affected in the current climate of social distancing and closure of education facilities. Psychologically, an exposure to potentially life-threatening traumatic experiences is particularly of concern to predisposed vulnerable individuals who struggle to adapt to this rapidly evolving situation and may experience varying degrees of adjustment disorders and stress-response symptoms. Frontline health care professionals are at greater risk of compromised mental and physical health as they have experienced a loss of a sense of agency by bearing witness to the fragility and suddenness of this crisis. In a progressive move to support existing mental health services, the Australian Government allocated $1.1 billion in funding for the implementation of coronavirus-wellness call centres, domestic abuse programmes and core mental health services (Office of PM, 2020b). This was actioned due to the avalanche of job losses and claustrophobic experiences of families being ‘locked up’ inside their houses that may lead to a spiral in distress, domestic violence and surge in mental illness and suicides.

## Cultural impact

Culture is one of the most fundamental ways in which people shape their world in a unique expression of cultural imprint. Culture is a broad and multi-layered concept that can be defined in many different ways and according to various disciplines. In this paper, we consider culture as a collective phenomenon that characterises an ascriptive heterogeneous group of people who share a set of defining values, attitudes, norms, symbols and customs. The Dutch social psychologist, Geert Hofstede, developed a framework of cultural dimensions in the context of anthropological and societal factors of organisations (Hofstede & Bond, 1984; Hofstede, 2011). Hofstede identified culture as a multi-dimensional psychosocial construct but later in his research redefined dominant cultural value systems using McCrae and Costa’s five-factor model of personality, noting that mean personality traits’ scores from 33 countries were significantly correlated with corresponding cultural dimensions’ scores (Hofstede & McCrae, 2004). Finnie (2020) mapped four of these cultural dimensions (namely, hierarchy or power distance, individualism, uncertainty avoidance and time orientation) in her cross-comparison analysis of national response to COVID-19 in Australia, China, South Africa, Italy and USA. Her article pointed to a definitive link between culture and behavioural responses while potentially implicating mental health outcomes and resilience.

Historically, Australia has prided itself in its multiculturalism; however, there has been evidence of an increase in racial microaggressions and xenophobia during this pandemic. The Australian senator, Pauline Hanson founder of the right-wing party ‘One Nation’, has been quoted: ‘any attempts to attack or criticise people for referring to COVID-19 as ‘Chinese Virus’ should be pushed back’ (Fang *et al*. 2020). Australian paper *Herald Sun* came in for criticism over framing the coronavirus on its front page with a Communist star and a surgical mask while alluding to China’s native pandas in the provocative headlines of ‘Chinese virus pandamonium’ (Argoon & McArthur, 2020), on the same day, *The Daily Telegraph* published an article ‘China kids stay home’ (Armstrong & Hildebrandt, 2020). One outcome from this divisive provocation by two mainstream Australian newspapers resulted in a petition by 93 000 signatories demanding an apology. These examples resonate with Wen’s study suggesting that in the current social climate of misleading and culturally insensitive media coverage of COVID-19; Chinese people living in Australia and overseas could suffer increased mental health problems (Wen *et al*. 2020). Wen’s study emulated an earlier study by Rodriguez-Seijas *et al*. (2015) who established an association between experiences of perceived racial discrimination and each of 12 common psychiatric diagnoses based on a nationally representative sample (*n* = 5191) of African American and Afro-Caribbean adults in USA.

## Australian indigenous communities

**Fig. 1.**
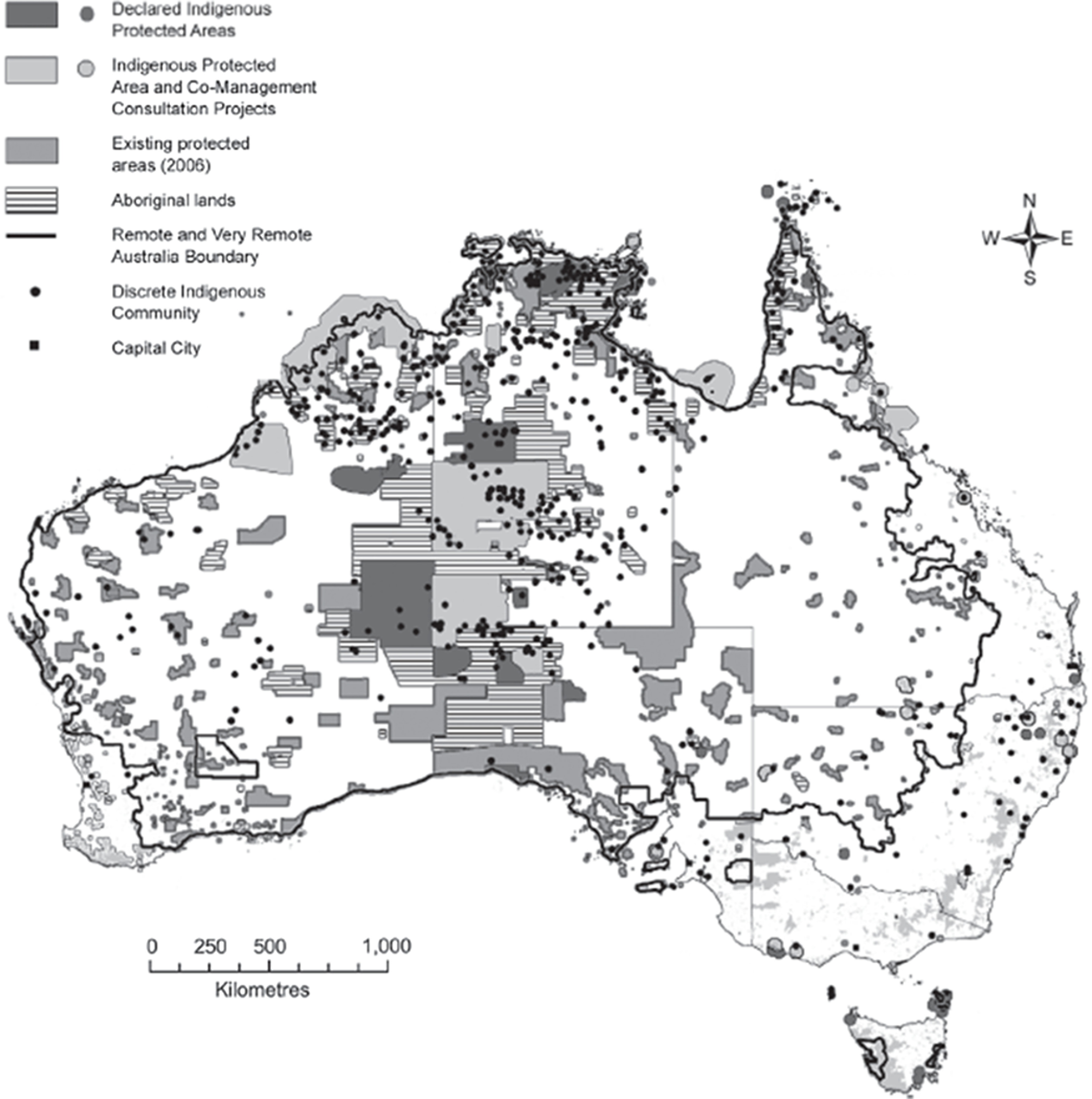
Indigenous protected areas and discrete Indigenous communities (from Altman & Fogarty, 2010).

Current literature suggests that COVID-19 acquisition rates and health outcomes vary according to age, sex, race, ethnicity and underlying health status (Richardson *et al*. 2020). Remote Aboriginal Australian Communities have been identified as a high-risk subpopulation in view of their unique vulnerabilities owing to their compromised health status, in addition to historical, systemic and cultural factors. Furthermore, there is a particular fear of endangering Aboriginal Australian Elders. These communities are represented by approximately 150 thousand settlers across the width of Australian continent (Australian Bureau of Statistics, 2016 and Fig. 1) who have been able to maintain their languages and traditions and engage in cultural practices that are distinctly indigenous. These groups have strong attentiveness of the ‘land’ and keep much closer involvement within their smaller communities (Miller, 2018) that vary in size between 100 and 1000 people. In these very remote communities, Indigenous Australians viewed as high collectivists (Miller, 2018), and unlike the rest of Australia, are almost beyond the reach of the state with many embracing ‘the art of not being governed’ (Scott, 2009; Altman & Fogarty, 2010) and mistrusting services outside of their locale (Sue & Sue, 1990).

People with underlying medical conditions are known to be at greater risk from COVID-19 – and diabetes, respiratory and cardiovascular conditions are more prevalent among Indigenous Australians. High reported smoking rates are also concerning as smoking makes individuals more susceptible to a respiratory infection – 37% of Indigenous young people over the age of 15 smoke (Commonwealth of Australia, 2020). The standardised rates (95% CI) of ‘any mental disorder’ in Indigenous adults, as ascertained in a recent cross-sectional study (*n* = 544), were 4.2-fold higher than among non-indigenous comparison group, ranging between 38.8% and 47.7% (Nasir *et al*. 2018). Another concerning statistic comes from a survey of Indigenous young people (Blair *et al*. 2005); in this sample, 9% of females and 4.1% of males reported that they had attempted suicide in the prior 12 months.

Indigenous Australians’ tendency to handle emotional distress on their own and strong reliance on spirituality to help ‘weather the storm’ may explain why they are less inclined to seek treatment from mental health specialists. On the other hand, clinician bias and stereotyping are well-recognised universal cultural factors that adversely affect service delivery. From cultural beliefs’ perspective, Indigenous groups may not adhere to COVID-19’ directive, as they may not comply with new rules of social distancing due to their traumatic past as they have been subjected to coercive and restrictive measures. Their unique connection to the land and reliance on their community has been described as potential protective factor (Nasir *et al*. 2018), but at the time of pandemic, this insularity of existence may prevent Indigenous Australians from receiving appropriate help. They may also find logistic difficulties in accessing care, as their designated medical workforce consists mainly of fly-in-fly-out staff, from Australia and New Zealand who are hindered by imposed quarantine restrictions. These concerns are further supported by grim reality of waiting times; in a study of rural and remote health conducted by the Royal Flying Doctor Service of Australia, some of survey respondents waited for up to 6 days or longer to access a doctor (Bishop *et al*. 2017). Telehealth services, while widely available, could be problematic due to communication and language barriers especially for those who speak English as a second, third or fourth language.

## Personal reflections

### ‘On fears among us’

In his famous essay on ‘Panopticism’, French philosopher Michel Foucault (Foucault, 1977) wrote that ‘the plague is met by order’. Similarly, we may experience that the COVID-19 outbreak is dictating a ‘new (world) order’ that comes with its own signifiers, its wartime language and, reminiscent of World War II, images of blackened boarded up windows on affected cruise ships. Are we being mobilised as frontline storm troopers, suddenly on lockdown, the question is are we advancing or retreating? Australia’s new biosecurity laws ensure containment, compliance and lack of movement that restrict our freedoms, which without a virus would be considered draconian. We identify with our First Nation citizens as we see these measures as coercive but are ready to show compliance amidst the pandemic due to medical rationalisation; nevertheless, abandoning normal legal channels for decision making and embracing these anti-democratic measures in the name of war against an invisible enemy, a virus.

Immediate implementation of biosecurity rules and regulations on social distancing are being made law, but as we cannot see our enemy what are we distancing ourselves from? If human avoidance is now part of survival, will we develop new phobias about closeness and touching when the pandemic passes? Without even realising, we are building a ‘*panopticist*’ prison of self-containment with increased surveillance by our governments and its loyal people. We are suspiciously watching each other through empty supermarket shelves with an eerie sense of paranoia that is palpable and unresolved. What if your unsuspecting neighbour has the disease or is a carrier? Whatever the individual cultural variations might be, an authoritarian trend in global politics is increasing while a democratic tradition appears to be in decline.

We now ask ourselves: could too much freedom create problems for humanity? It is worth considering the concept of *Jouissance* by French psychoanalyst Jacques Lacan (1969–1970) that drives repetition and relates to an idea of excess in life, or an enjoyment beyond pleasure, as when we have exponential pleasure it results in *unpleasure*, a torture. In reflecting upon the state of our planet, the land we coexist on, it is apparent that all was not well before the pandemic as we have exceeded our potential to sustain ourselves as a species by destroying our very existence via our capacity to consume. There are reports that skies and environment became clearer as tourists’ numbers dramatically dropped and car, bus and flight travel has reduced. We have been asked to stop, to stop buying so much, stop dumping so much, stop being cruel and stop being free. Extreme containment creates problems, but so does too much freedom, which further supports Lacan’s theoretical premise. Rather than a measured, logical response of limiting environmental exposure or stopping what we did before, a reflexive reaction has been observed. A universal desire for a panacea in the form of an imaginary, untested vaccine has manifested. This vaccine may reconcile the psychic conflict of meeting our *narcissistic demand*, so that we succeed at a achieving a state of a *Jouissance of excess*, feed our *compulsion to repeat* and do it all over again.

### On cultural awareness and resilience

What we are facing is daunting, and it is crucial to note that we are viewing the world through our own lens, own cultural background, cultural norms, values and biases. Between two collaborators of this paper, we have benefited from exposure to cultural traditions in a plethora of countries across all continents: Russia, Ireland, Africa, Europe, USA, South East Asia and Australia. Hence, it is our shared view that adaptive capacity to bounce back and not to succumb to the negative effects of any threatening situation are influenced by cultural factors that interact with host of other determinants at the level of biological underpinning, familial patterns of behaviour and individual psychosocial factors. According to De Vaus et al. (2017), the way Easterners and Westerners think about negative emotions could be traced back to two cultural dimensions: holistic and analytic systems of thought. According to their model, Easterners are more resilient in absorbing relatively high levels of negative emotions without experiencing distress or becoming mentally unstable. Their resilience is rooted in what we increasingly recognise as core Bateman and Fonagy’s Mentalization – Based skills of fostering curiosity, awareness and acceptance (rather than avoidance and fear) that allow for better contextual understanding of the problem and greater flexibility in the use of emotion regulation strategies (Bateman & Fonagy, 2013). Mentalization fosters resilience by furnishing an individual with mental abilities that have an elastic band quality, that is, being able to bend without breaking and could even provide an opportunity for growth at the time of crisis. Holistically minded individuals are also more willing to embrace the possibility of contradiction and change and to experience negative emotions as less intrinsically tied to the individual self than in analytic cultures.

Conversely, the West places a relatively high value on positive emotions, thus one experiences greater discomfort when faced with unwanted negative emotions. The pursuit of happiness that is being propagated as a goal in the Western cultures could be blinding for an individual as it diverts from more meaningful long-term values that may be protective at times of adversities. That’s why many ‘inspired’ organisations cultivate the philosophical platform of ‘meaning over happiness’ when promoting mental wellness and protecting longevity of their workforce (Dwyer *et al*. 2017).

During stressful times, people revert to their traditional values, their comfortable cultural space and adapt less, as their neuroplasticity and ability to form new neurolinguistic pathways are greatly reduced. Based on the premise that ‘all disasters are local’, we need to ensure a ‘whole of society’ versus a ‘whole of government’ response. Australia’s model of cultural awareness will need to evolve, from reactionary to more reflective, post COVID-19 pandemic to best serve our multicultural, inclusive and integrated society.
